# Application of IVIM-DWI in Detecting the Tumor Vasculogenic Mimicry Under Antiangiogenesis Combined With Oxaliplatin Treatment

**DOI:** 10.3389/fonc.2020.01376

**Published:** 2020-08-18

**Authors:** Jianye Liang, Zhipeng Li, Jing Li, Chuan Peng, Wei Dai, Haoqiang He, Sihui Zeng, Chuanmiao Xie

**Affiliations:** ^1^Department of Medical Imaging, Sun Yat-sen University Cancer Center, State Key Laboratory of Oncology in South China, Collaborative Innovation Center for Cancer Medicine, Guangzhou, China; ^2^Medical Imaging Center, The First Affiliated Hospital of Jinan University, Guangzhou, China

**Keywords:** vascular normalization, vasculogenic mimicry, epithelial–mesenchymal transition, intravoxel incoherent motion diffusion-weighted imaging (IVIM-DWI), MRI, colon cancer

## Abstract

**Objectives:** This study aimed to detect the time window of vascular normalization during anti-vascular treatment using intravoxel incoherent motion diffusion-weighted imaging (IVIM-DWI). Simultaneously, we evaluated the tumor invasiveness and vasculogenic mimicry and performed synthetic assessment of treatment efficacy of angiogenesis inhibitor combined with conventional chemotherapy using IVIM-DWI.

**Materials and Methods:** HCT116 cells were subcutaneously administered into the right flank of BALB/C nude mice to build a colon cancer xenograft model. Thirty-two tumor-bearing mice were randomly divided into four groups and intraperitoneally administered with normal saline (Group A or control group), bevacizumab (Group B), oxaliplatin monotherapy (Group C), and oxaliplatin combined with bevacizumab (Group D). The IVIM-DWI was performed on days 0, 3, 6, 9, 12, and 15 after the treatments. Another 51 tumor-bearing mice were included in the pathological examinations. α-Smooth muscle actin (SMA) and CD31 double-staining, periodic acid-Schiff (PAS) and CD31 double-staining, hematoxylin and eosin (HE), Ki-67, and E-cadherin staining were performed. The tumor growth and dynamic change of each parameter were noted.

**Results:** The mice in Group D manifested the smallest tumor volume and highest tumor inhibition rate. Microvessel density was significantly decreased but accompanied by increased vasculogenic mimicry after antiangiogenic treatment. The trend was reversed by oxaliplatin treatment. Treated with bevacizumab, the vessel maturity index shared a similar trend with D^*^ and *f*-values during days 3–12, which slowly increased from days 0 to 9 and then decreased briefly. *D*-value significantly correlated with vasculogenic mimicry and Ki-67, while D^*^ and *f*-values showed positive correlations with microvessel density and E-cadherin, an indicator of epithelial–mesenchymal transition.

**Conclusion:** Oxaliplatin performed an inhibited effect on vasculogenic mimicry. Bevacizumab can enhance the tumor chemotherapy through vascular normalization within a transient time period, which can be detected by IVIM-DWI. D^*^ and *f*-values are able to predict the tumor invasiveness while *D* is superior in reflecting vasculogenic mimicry and Ki-67 expression during antitumor treatment.

## Introduction

Tumor vascularization plays an important role in tumor growth, invasion, and metastasis. The lack of adequate oxygen supply for tumor growth will induce the formation of a hypoxia microenvironment. In order to counteract the severe condition, the tumor cells and tumor microenvironment will upregulate the expression of vascular endothelial growth factor (VEGF) and hypoxia-inducible factor 1α (HIF-1α) and subsequently perform a series of adaptive changes. Initially, a great amount of newborn vessels are formed, with high leakage due to the absence of smooth muscle cells and pericyte coverages, and incomplete basement membrane. The immature vessels deliver inadequate nutrition and oxygen but elevate the interstitial fluid pressure, which will aggravate the tumor hypoxia and lead to tumor cell transformation into an aggressive phenotype. Epithelial–mesenchymal transition (EMT) will be induced by activating HIF-1α (1), and the tumor cells will lose their epithelial phenotypes but acquire mesenchymal phenotypes with migration ability, enhancing the tumor invasiveness and the metastatic potential. E-cadherin, which is mainly expressed on the epithelial cell membranes, is proved to be most correlated with tumor invasiveness and metastasis and considered as an important indicator of EMT (2, 3).

Antiangiogenic therapy has been widely used in the clinical treatment of various solid tumors. It is a promising therapy that targets the VEGF which plays an important role on the neovasculature formation. Jain (4) proposed the concept of “normalizing tumor vasculature” in 2001, which is dedicated to improve the blood supply and relieve tumor hypoxia by repairing immature tumor vessels. This could further relieve the tumor invasiveness and improve treatment response (5). However, antiangiogenesis monotherapy seemed inadequate since it neither enhanced the tumor response rate nor significantly prolonged the overall survival in most tumors (6), largely due to rapid revascularization in new angiogenic modes including glomeruloid angiogenesis, vessel co-option, intussusceptive microvascular growth, looping angiogenesis, and vasculogenic mimicry (VM) via alternative proangiogenic signaling pathways (7, 8). A previous study has indicated that vessel co-option is a significant mechanism of resistance to antiangiogenic therapy (9). VM is another patterned structure independent of sprouting angiogenesis. Highly invasive and plastic cancer cells directly envelop the vessel-like channels by imitating endothelial cells but negative for characteristics of endothelial cell marker CD31 or CD34. It has been reported to have a close relationship with tumor grade/differentiation, invasion and metastasis, and poor clinical prognosis (10, 11). However, few studies have prompted the relationship between VM and treatment efficacy in a combined therapy setting, especially the agents targeting VM. Zinc-finger E-box binding homeobox 1 (ZEB1) is another important inducible factor of EMT in many carcinoma cells including colorectal cancer (12). Liu et al. (13) reported that ZEB1 can promote VM formation by inducing EMT, revealing the close relationship between EMT and VM in colorectal cancer, which should be further confirmed.

Oxaliplatin is the third-generation platinum anticancer drug. It has an active antitumor effect against a variety of tumors and performs a synergistic effect with 5-fluorouracil (14). However, clinical studies demonstrated inconsistent efficacy of oxaliplatin combined with bevacizumab in the treatment of advanced colorectal cancer with unclear anticancer mechanisms. A phase III randomized controlled trial demonstrated that adding bevacizumab to oxaliplatin-based chemotherapy cannot prolong disease-free survival in resected stage III colon cancer but performed a potential detrimental effect in these patients (15). However, a meta-analysis suggested that oxaliplatin combined with bevacizumab as a maintenance therapy can significantly improve the overall response rate of patients with advanced metastatic colon cancer and recommended as a first-line treatment option for these patients (16). These inconsistent results arouse our interests to further explore their efficacy and mechanism by pathology and imaging.

Monitoring the tumor microenvironment is an important direction of current antitumor therapy, including the degree of tumor hypoxia, EMT, and various compensatory angiogenesis pathways, which have close relationships with each other and determine the efficacy of chemoradiotherapy or immunotherapy. Currently, how to effectively monitor the dynamic change of EMT and VM as well as vascular normalization and therapeutic efficacy is the key point of this research. Although dynamic contrast-enhanced magnetic resonance imaging (DCE-MRI) is the gold standard for evaluating vascular perfusion and permeability in imaging, continuous enhanced imaging in a short time requires multiple injections of contrast agent, which poses a certain risk to the organisms. Intravoxel incoherent motion diffusion-weighted imaging (IVIM-DWI) is a noninvasive technique which was first proposed by Le Bihan et al. (17) in 1986. It can sensitively reflect the tumor cellularity and microcirculation perfusion in tissues without the need of contrast agent (18), allowing for longitudinal monitoring of cellular and hemodynamic changes. We attempted to perform the IVIM-DWI to assess vascular normalization during anti-vascular treatment in the study. Moreover, we monitored the tumor microenvironment changes including tumor invasiveness and VM by correlating the imaging parameters with pathological parameters, investigated the mechanism of bevacizumab combined with oxaliplatin in a colon cancer xenograft model, and evaluated its efficacy.

## Materials and Methods

### Cell Culture

We obtained the human colon cancer HCT116 cell line from the Pharmaceutical College of Jinan University. They were cultured in Dulbecco's modified Eagle's medium (DMEM, Gibco) supplemented with 10% fetal bovine serum and 1% penicillin/streptomycin at 37°C in 5% CO_2_ atmosphere.

### Tumor Model and Grouping

The procedure for animal experiment was approved by the Institutional Animal Ethics Committee of Jinan University. Relevant recommendations regarding animal ethics and welfare were strictly followed. A total of 100 female BALB/c nude mice (aged 5–8 weeks, weighed 17–22 g) were purchased from Beijing Vital River Laboratory Animal Technology Corporation (Beijing, China) and kept in specific pathogen-free conditions. HCT116 cells (0.2 ml/mouse, 1 × 10^6^/ml) mixed with 10 ml of Matrigel were subcutaneously injected into the right flank of the mice for creating colon cancer-bearing mice. At last, 83 tumor-bearing mice were selected for imaging (*n* = 32) and pathological examinations (*n* = 51). The mice for the MRI examinations were randomly divided into four groups and then intraperitoneally injected with normal saline (Group A or control group), bevacizumab (5 mg/kg; Group B), oxaliplatin (4 mg/kg; Group C), and oxaliplatin combined with bevacizumab (Group D) on days 1, 4, 7, 10, and 13. The tumor volume was calculated using the following formula: (*a*^2^ × *b* × 0.5) mm^3^, where *a* refers to the smaller diameter and *b* is the diameter perpendicular to *a*, measured with a slide caliper (19). The tumor inhibition rate refers to (tumor size of untreated mice—tumor size of treated mice)/tumor size of untreated mice × 100%.

### MRI Examinations

The MRI examination was conducted on a 1.5 T Signa HDxt superconductor MR system (GE Healthcare, Milwaukee, WI) with an eight-channel wrist coil. The four groups of mice were intraperitoneally administered with 0.1% pentobarbital solution and scanned in the supine position prior to and on days 3, 6, 9, 12, and 15 after therapy (5). The interval of 3 days between the scans was made under the consideration for the relatively long period of recovery from anesthetizations, the high survival rate for the nude mice, and as well as the slow efficacy of bevacizumab to manifest antiangiogenic effects during the scan period. Fast spin-echo sequence (FSE) was applied for T1-weighted imaging (T1WI). Relevant parameters included repetition time/echo time (TR/TE) of 540/14.7 ms, slice thickness of 2 mm, slice gap of 0.2 mm, field of view (FOV) of 5 × 5 cm^2^, matrix size of 192 × 160, number of excitations (NEX) of 2. T2WI was performed with fast recovery FSE and TR/TE of 2,140/82.3 ms. The other parameters were in accord with T1WI. The IVIM-DW MR images were obtained using a single-shot, echo-planar imaging pulse sequence with chemical shift-selective saturation technique for fat suppression. Three orthogonal directions were set as the diffusion gradients with 13 *b*-values: 0, 25, 50, 75, 100, 150, 200, 400, 600, 800, 1,000, 1,200, and 1,500 s/mm^2^, TR/TE of 4,200/101.7 ms, NEX of 3 for each *b*-value, matrix size of 96 × 128, and FOV of 7.0 × 5.6 cm^2^. This sequence took 7 min 20 s, with six slices covering the whole tumor.

### Image Post-processing

All IVIM-DWI data were transferred to a dedicated post-processing workstation (AW4.5, GE Health care, USA) using the Functool-MADC software. The equation of the bi-exponential model was expressed as SI/SI_0_ = (1 – *f*) × exp(–*bD*) + *f* × exp(–bD^*^). SI_0_ refers to the mean signal intensity of the region of interest (ROI) for a *b*-value of 0 s/mm^2^, while SI refers to the signal intensity for higher *b*-values. *B*-value means the diffusion sensitivity coefficient. *D*-values represent the pure diffusion of water molecule, also known as true diffusion coefficient. D^*^ values refer to pseudo-diffusion, which calculate the amount of microcirculation perfusion. *F*-value is the perfusion fraction, standing for the percentage of microcirculation perfusion among the signal decay from the total diffusion effect. A segmented method was used to fit the bi-exponential IVIM model. First, *b* < 200 mm^2^/s was referred to as low *b*-value, mainly reflecting the pseudo-diffusion. The data in this range were fitted to the bi-exponential model for acquiring D^*^ and *f*-values. Then the data of *b*-values higher than 200 mm^2^/s were used to obtain *D*-value using a mono-exponential model because the pseudo-diffusions from blood flows were negligible in this region (20). Therefore, the mono-exponential model was expressed as SI/SI_0_ = exp(–*bD*) since the *f* and D^*^ values verge to zero at the *b*-values higher than 200 mm^2^/s. Structural T2WI was referenced to plot three ROIs at the largest cross section and adjacent slices of the tumor. The ROI was also indicated in the MRI figures with a white circle. We averaged the three ROIs as the final values.

### Histological Analysis

Out of the 51 mice that were included for pathological analysis, three of them were served for baseline histological analysis (day 0), and for the other 48 mice, each three were sacrificed and sampled on days 3, 6, 9, and 12 for each subgroup of histological analysis. Besides, the mice for the MRI examinations were all sacrificed on day 15. Their tumor samples were regarded as the pathological results. Hematoxylin and eosin (HE) staining, Ki-67 and E-cadherin immunofluorescent staining, periodic acid-Schiff (PAS) and CD31 immunohistochemical double-staining, and α-smooth muscle actin (SMA) and CD31 immunofluorescent double-staining were performed to analyze the pathological changes. The antibodies were all purchased from Servicebio Technology Co., Ltd. (Wuhan, China). Ki-67 is a proliferation marker reflecting the degree of proliferation activity of tumor cells. VM can be recognized by positive PAS staining and negative CD31 staining, a marker of endothelial cells (21). The tumor sample was fixed in 4% paraformaldehyde for 24 h. They were embedded in paraffin and sectioned at 5-μm thickness for subsequent staining. An Olympus BX 53 microscope was used to observe the staining slices. The microvessel density (MVD) was calculated using the “hot spot” method (22). The areas of highest neovascularization was scanned throughout the tumor sections at low power (40×) by identifying the areas with the highest number of discrete microvessel staining for CD31 (brown), and individual microvessels were manually counted on a 200× field. Image-Pro Plus 6.0 software (Media Cybernetics, MD, USA) was used to detect and measure the percentage of the positively stained cells by different antibodies on high magnification (×200). α-SMA is an indicator of pericyte coverage and can be used to calculate vessel maturity index (VMI), a ratio of positive α-SMA area to CD31 area (23).

### Statistical Analysis

We used SPSS 13.0 software (IBM Corporation, Chicago, IL, USA) to calculate statistical results. The data distribution type was confirmed using Kolmogorov–Smirnov test. The numeric result with normal distribution was shown as the mean value with standard deviation (SD). The difference within and between groups were compared by one-way analysis of variance (ANOVA) with Student–Newman–Keuls q test as a *post hoc* test. The correlation strengths between imaging parameters and pathological results on day 15 were calculated using Pearson correlation analysis. *P* < 0.05 was regarded a statistical difference. A Pearson coefficient larger than 0.8 was considered highly correlated, while the coefficient between 0.5 and 0.8 was considered moderately correlated. The line chart and scatter chart were plotted with GraphPad Prism 5.01 (GraphPad Software Inc., San Diego, CA).

## Results

### Treatments on Tumor Growth

No mice died or showed obvious abnormal reactions during the treatment. An obvious inhibitory effect on tumor growth was shown as early as 6 days after the treatment with a much slower growth rate in tumor volume during treatment. The mice treated with the combination therapy manifested the smallest tumor volume and highest tumor inhibition rate at the final time point. The mice treated with oxaliplatin showed smaller tumor volume than that with bevacizumab and compared with the control group. The tumor inhibition rates on day 15 were 35.5%, 49.6%, and 68.6% in Groups B, C, and D, respectively.

### MRI Results

**Figure 1:**
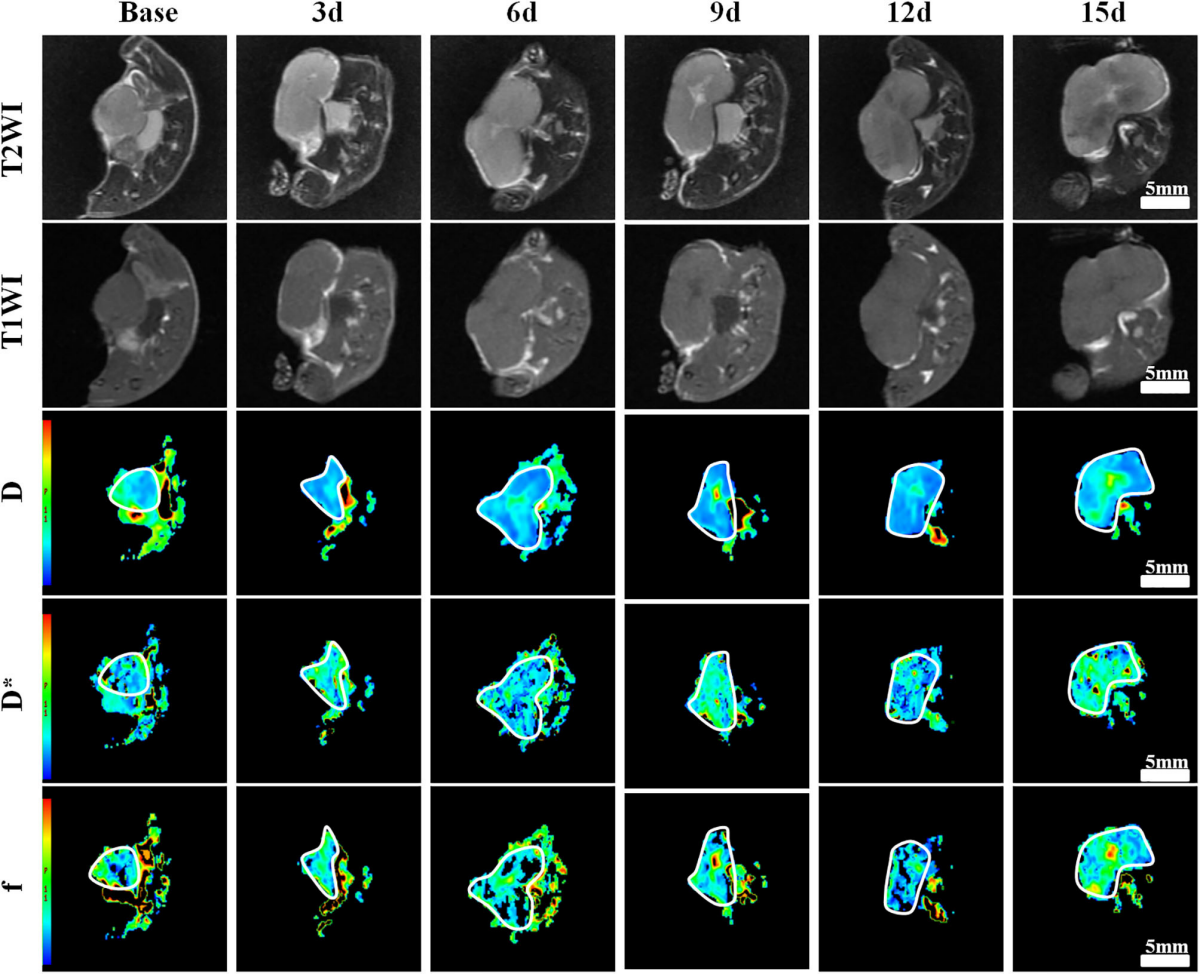
Axial T1-weighted imaging (T1WI), T2WI, and pseudocolor maps of D, D*, and *f*-values at different time points treated with bevacizumab and oxaliplatin (Group D). *D*-value is the pure diffusion of water molecule. D* value refers to perfusion-related diffusion. *F*-value is the perfusion fraction. The white circles plot the tumor area. The tumor showed a much slow growth during the 3rd−15th days. A small patch of high signal was observed in the tumor core on T2WI as early as 6 days after treatment. Color ranging from blue to red represents values ranging from low to high. *D*-value increased significantly after 6 days and reached a peak on day 15. D* and *f*-values increased to a peak on day 9, and then fell back until day 15.

**Figure 2:**
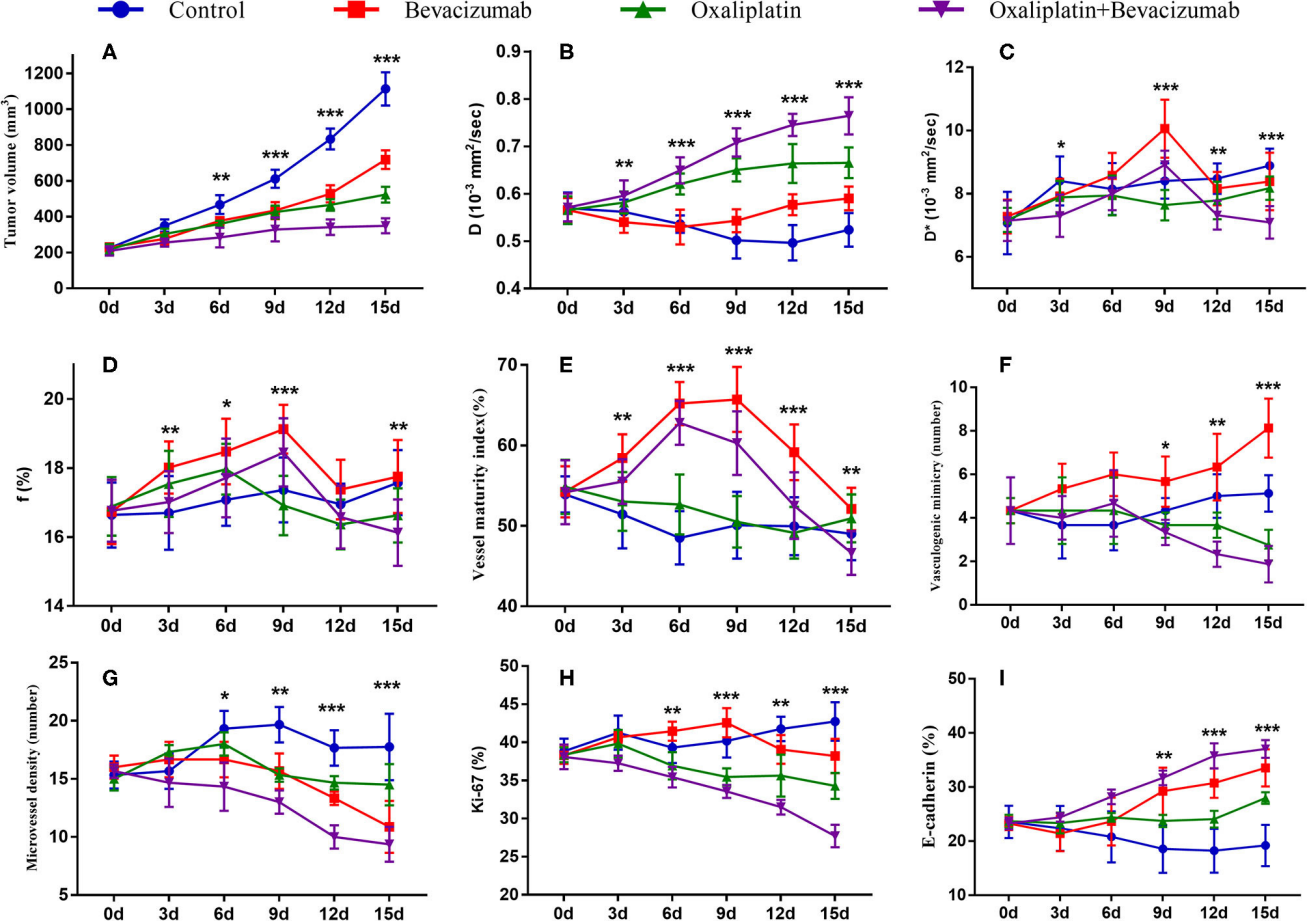
Longitudinal monitoring of intravoxel incoherent motion diffusion-weighted imaging (IVIM-DWI) and pathological parameters as well as tumor growth in the four groups. The data point indicated mean and standard deviation. *P < 0.05, **P < 0.01, and ***P < 0.001 were generated from comparisons between the four groups at each time point using one-way analysis of variance. Graphs show trends for tumor growth **(A)**, and MRI parameters–D value **(B)**, D^*^ value **(C)**, and f value **(D)**, and vessel maturity index **(E)**, vasculogenic mimicry **(F)**, microvessel density **(G)**, Ki-67 index **(H)** and E-cadherin **(I)**.

One group of representative conventional T1WI, T2WI, and pseudo-color maps of D, D^*^, and *f*-values in Group D is manifested in Figure 1. The mean values and line charts of Groups A, B, C, and D are manifested in Tables 1–4 and Figure 2.

**Table 1:** IVIM-DWI and pathological parameters in different time points from Group A.

**Group A**	**Base**	**Day 3**	**Day 6**	**Day 9**	**Day 12**	**Day 15**	***F***	***P***
*D* (10^−3^ mm^2^/s)	0.570 ± 0.033	0.562 ± 0.026	0.536 ± 0.018	0.502 ± 0.038	0.497 ± 0.037	0.524 ± 0.035	6.971	0.001
D* (10^−3^ mm^2^/s)	7.07 ± 0.99	8.40 ± 0.78	8.15 ± 0.82	8.40 ± 0.56	8.48 ± 0.48	8.89 ± 0.53	5.937	0.001
*f* (%)	16.6 ± 0.9	16.7 ± 1.1	17.1 ± 0.7	17.4 ± 0.9	17.0 ± 0.6	17.6 ± 0.9	1.377	0.252
VMI (%)	53.9 ± 2.3	51.4 ± 4.2	48.5 ± 3.3	50.1 ± 4.2	50.0 ± 3.6	49.0 ± 3.3	2.446	0.049
MVD	15.3 ± 1.2	15.7 ± 1.5	19.3 ± 1.5	19.7 ± 1.5	17.7 ± 1.5	17.8 ± 2.9	2.098	0.116
VM	4.3 ± 1.5	3.7 ± 1.5	3.7 ± 1.2	4.3 ± 0.6	5.0 ± 1.0	5.1 ± 0.8	1.426	0.265
Ki-67 (%)	38.8 ± 1.6	41.3 ± 2.2	39.3 ± 2.0	40.2 ± 2.2	41.8 ± 1.6	42.7 ± 2.5	2.068	0.12
E-cadherin (%)	23.6 ± 3.0	22.4 ± 4.2	20.8 ± 4.7	18.6 ± 4.4	18.2 ± 4.1	19.2 ± 3.8	0.962	0.468
Tumor volume (mm^3^)	224.9 ± 23.5	350.5 ± 34.1	467.8 ± 52.4	611.8 ± 50.5	834.0 ± 57.9	1,113.8 ± 92.4	273.964	0.001

*Repeated measures analysis of variance was used to generate the P-value. IVIM-DWI, intravoxel incoherent motion diffusion-weighted imaging; VMI, vascular maturity index; MVD, microvessel density; VM, vasculogenic mimicry*.

**Table 2:** IVIM-DWI and pathological parameters in different time points from Group B.

**Group B**	**Base**	**Day 3**	**Day 6**	**Day 9**	**Day 12**	**Day 15**	***F***	***P***
*D* (10^−3^ mm^2^/s)	0.566 ± 0.026	0.540 ± 0.023	0.530 ± 0.037	0.543 ± 0.024	0.577 ± 0.022	0.590 ± 0.025	6.321	0.001
D* (10^−3^ mm^2^/s)	7.28 ± 0.54	7.93 ± 0.55	8.58 ± 0.71	10.06 ± 0.92	8.16 ± 0.53	8.39 ± 0.91	13.649	0.001
*f* (%)	16.7 ± 0.9	18.0 ± 0.8	18.5 ± 1.0	19.1 ± 0.7	17.4 ± 0.9	17.8 ± 1.1	7.192	0.001
VMI (%)	54.2 ± 3.2	58.5 ± 2.9	65.2 ± 2.7	65.7 ± 4.0	59.2 ± 3.5	52.1 ± 2.6	24.228	0.001
MVD	16.0 ± 1.0	16.7 ± 1.5	16.7 ± 1.5	15.7 ± 2.1	13.3 ± 0.6	10.9 ± 2.2	9.397	0.001
VM	4.3 ± 1.5	5.3 ± 1.1	6.0 ± 1.0	5.7 ± 1.2	6.3 ± 1.5	8.1 ± 1.4	5.606	0.003
Ki-67 (%)	38.3 ± 1.1	40.7 ± 0.7	41.5 ± 1.3	42.6 ± 1.9	39.1 ± 1.9	38.2 ± 2.2	3.706	0.019
E-cadherin (%)	23.3 ± 1.2	21.4 ± 3.2	23.6 ± 4.4	29.2 ± 4.4	30.7 ± 2.7	33.6 ± 3.4	9.054	0.001
Tumor volume (mm^3^)	229.5 ± 22.7	376.9 ± 32.1	377.1 ± 20.0	434.8 ± 47.1	528.3 ± 47.7	718.5 ± 52.2	166.808	0.001

*Repeated measures analysis of variance was used to generate the P-value. IVIM-DWI, intravoxel incoherent motion diffusion-weighted imaging; VMI, vascular maturity index; MVD, microvessel density; VM, vasculogenic mimicry*.

**Table 3:** IVIM-DWI and pathological parameters in different time points from Group C.

**Group C**	**Base**	**Day 3**	**Day 6**	**Day 9**	**Day 12**	**Day 15**	***F***	***P***
*D* (10^−3^ mm^2^/s)	0.566 ± 0.029	0.582 ± 0.023	0.620 ± 0.022	0.650 ± 0.024	0.664 ± 0.041	0.665 ± 0.032	17.116	0.001
D* (10^−3^ mm^2^/s)	7.17 ± 0.38	7.89 ± 0.52	7.94 ± 0.63	7.64 ± 0.48	7.79 ± 0.59	8.17 ± 0.37	3.665	0.008
*f* (%)	16.9 ± 0.9	17.5 ± 1.0	18.0 ± 0.7	16.9 ± 0.9	16.4 ± 0.7	16.6 ± 0.8	4.181	0.004
VMI (%)	54.9 ± 3.4	53.0 ± 3.6	52.7 ± 3.7	50.5 ± 3.2	49.2 ± 3.2	50.9 ± 3.0	2.963	0.022
MVD	15.0 ± 1.0	17.3 ± 0.6	18.0 ± 1.0	15.3 ± 0.6	14.7 ± 0.6	14.5 ± 1.8	4.825	0.006
VM	4.3 ± 1.2	4.3 ± 1.5	4.3 ± 1.5	3.7 ± 0.6	3.7 ± 0.6	2.8 ± 0.7	2.762	0.045
Ki-67 (%)	38.4 ± 1.0	39.8 ± 1.8	36.9 ± 1.8	35.5 ± 1.1	35.6 ± 2.7	34.2 ± 1.7	5.634	0.003
E-cadherin (%)	23.7 ± 1.2	23.4 ± 1.9	24.4 ± 0.8	23.7 ± 1.1	24.1 ± 1.6	28.0 ± 1.1	11.67	0.001
Tumor volume (mm^3^)	218.5 ± 29.6	303.4 ± 30.1	360.5 ± 16.9	425.9 ± 35.8	465.9 ± 32.1	561.1 ± 36.1	125.249	0.001

*Repeated measures analysis of variance was used to generate the P-value. IVIM-DWI, intravoxel incoherent motion diffusion-weighted imaging; VMI, vascular maturity index; MVD, microvessel density; VM, vasculogenic mimicry*.

**Table 4:** IVIM-DWI and pathological parameters in different time points from Group D.

**Group D**	**Base**	**Day 3**	**Day 6**	**Day 9**	**Day 12**	**Day 15**	***F***	***P***
*D* (10^−3^ mm^2^/s)	0.571 ± 0.029	0.596 ± 0.032	0.650 ± 0.027	0.708 ± 0.030	0.745 ± 0.023	0.764 ± 0.039	54.02	0.001
D* (10^−3^ mm^2^/s)	7.14 ± 0.64	7.30 ± 0.67	7.99 ± 0.51	8.91 ± 0.45	7.32 ± 0.46	7.09 ± 0.51	13.301	0.001
*f* (%)	16.8 ± 0.9	17.0 ± 0.9	17.7 ± 1.1	18.5 ± 1.0	16.6 ± 0.9	16.1 ± 1.0	6.03	0.001
VMI (%)	54.2 ± 4.0	55.5 ± 2.8	62.8 ± 2.7	60.3 ± 3.9	52.5 ± 4.1	46.6 ± 2.7	22.366	0.001
MVD	15.7 ± 0.6	14.7 ± 2.1	14.3 ± 2.1	13.0 ± 1.0	10.0 ± 1.0	9.4 ± 1.5	13.281	0.001
VM	4.3 ± 1.5	4.0 ± 1.0	4.7 ± 1.5	3.3 ± 0.6	2.3 ± 0.6	1.9 ± 0.8	5.482	0.003
Ki-67 (%)	38.1 ± 1.6	37.3 ± 1.0	35.4 ± 1.4	33.6 ± 0.9	31.5 ± 1.0	27.7 ± 1.5	43.934	0.001
E-cadherin (%)	23.3 ± 1.1	24.4 ± 0.8	28.2 ± 1.3	31.7 ± 1.4	35.8 ± 2.3	37.1 ± 1.6	58.279	0.001
Tumor volume (mm^3^)	210.0 ± 25.9	256.0 ± 22.2	284.0 ± 54.6	32.3 ± 66.2	342.0 ± 43.1	349.9 ± 42.2	12.035	0.001

*Repeated measures analysis of variance was used to generate the P-value. IVIM-DWI, intravoxel incoherent motion diffusion-weighted imaging; VMI, vascular maturity index; MVD, microvessel density; VM, vasculogenic mimicry*.

On conventional images, the tumor showed slow growth with little tumor volume change during days 3–15 in Group D. Small patchy high signals indicating liquefactive necrosis were observed in the tumor core on T2WI as early as day 6 after the treatment in this group. For Groups B and C, the tumor volume moderately increased, and multiple scattered high signals were revealed on T2WI on days 12 and 15. For Group A, the tumor area showed a rapid increase with an obvious high signal on T2WI on day 12.

For the MR indicators, *D*-value showed a slow decline (*F* = 6.971, *P* < 0.001), while D^*^ (*F* = 5.937, *P* < 0.001) and *f*-values (*F* = 1.377, *P* = 0.252) gradually increased until day 15 in Group A, which may be caused by the physiological growth of the tumor with crowded cellularity and increased demand of blood supply over a long period of time.

For bevacizumab monotherapy, *D*-value slowly decreased over time and started to rise on day 6 (*F* = 6.321, *P* < 0.001), indicating that the restricted diffusion of water molecules was relieved due to the treatment. D^*^ (*F* = 13.649, *P* < 0.001) and *f*-values (*F* = 7.192, *P* < 0.001) gradually increased during day 0 to 9, representing an augment of tumor perfusion due to vascular normalization. They decreased between day 9 and 12, followed by a slight upward trend after day 12. The delayed antiangiogenic effect and the recession of vascular normalization may account for the subsequent changes on tumor perfusion.

For oxaliplatin monotherapy, *D*-value maintained a slow upward trend and was higher than those in Groups A and B (*F* = 17.116, *P* < 0.001), suggesting an improved treatment efficacy. D^*^ (*F* = 3.665, *P* = 0.008) and *f*-values (*F* = 4.181, *P* = 0.004) initially increased and then started to decrease on day 6. These may be a subsequent change due to tumor necrosis.

For combination therapy, *D*-value increased significantly after 6 days and reached a peak on day 15, which was also higher than that of the other groups. D^*^ (*F* = 13.301, *P* < 0.001) and *f*-values (*F* = 6.030, *P* < 0.001) increased to a peak on day 9 but lower than those of Group B, and then fell back until day 15. The trend indicated a similar anti-vascular effect as in Group B.

### Histological Results

**Figure 3:**
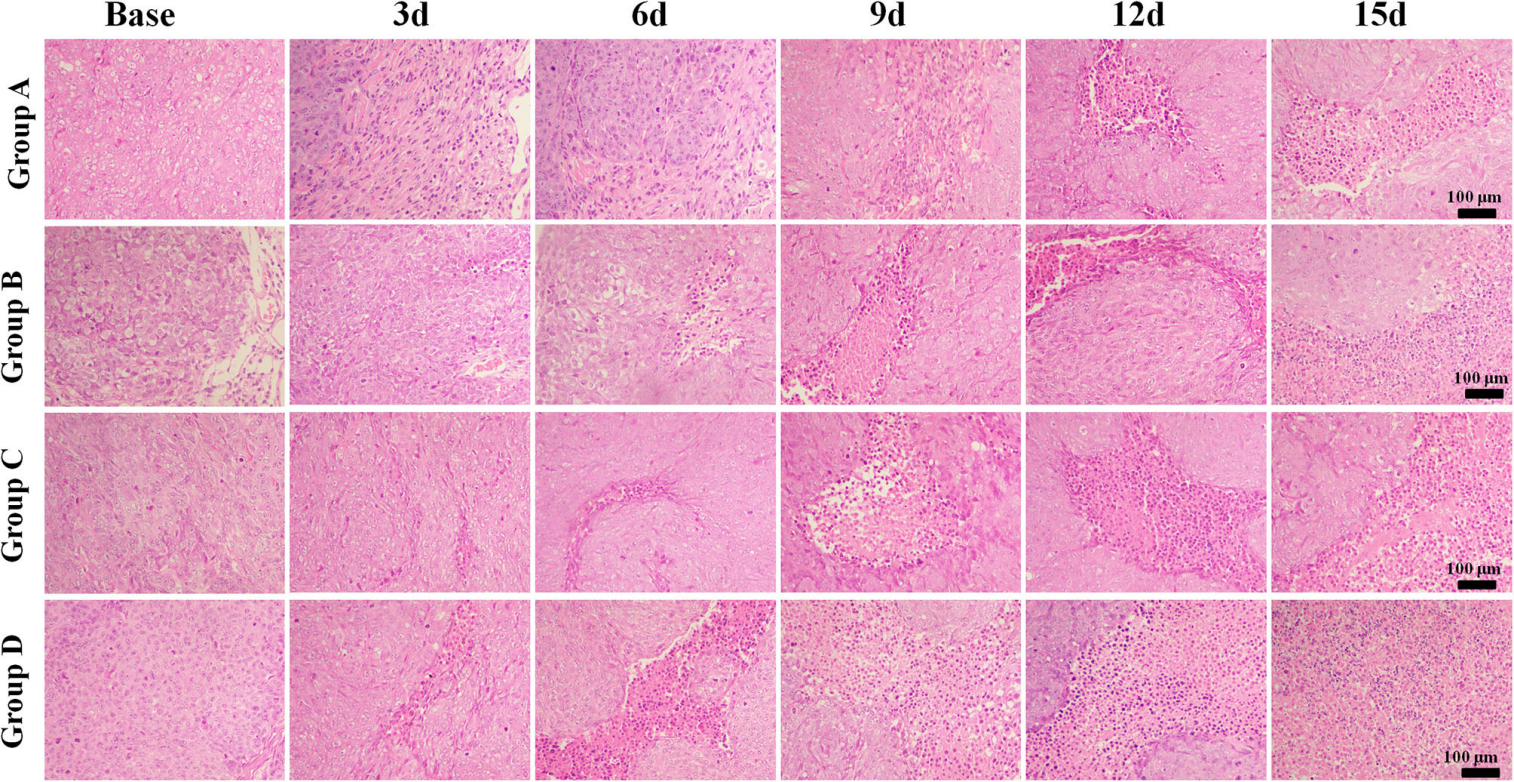
Hematoxylin and eosin staining (×100) of a representative tumor section from the four groups at different time points. More necrosis areas were observed in Group D and obvious on days 12 and 15. The tumor cells in Group A were dense. A few of necrosis areas were observed as the tumor grew. In Group B, a small amount of necrosis and cell apoptosis occurred in the tumor core. More karyopyknosis and nuclear fragmentation with homogeneous red-stained areas were observed in the later time points. In Group C, small patches of hemorrhage and necrosis appeared on day 3, which mainly distributed in the tumor core. More necrosis areas were observed in Group D, especially on days 12 and 15.

The mean values of pathological indicators in Groups A, B, C, and D at each time point are shown in Tables 1–4. Their line charts are manifested in Figure 2. The representative HE staining in Groups A, B, C, and D at six time points is shown in Figure 3.

The tumor cells in Group A were dense. A few necrosis areas were observed as the tumor grew. In Group B, a small amount of necrosis and cell apoptosis appeared in the tumor core. More karyopyknosis and nuclear fragmentation with homogeneous red-stained areas were observed in the later time points. In group C, small patches of hemorrhage and necrosis appeared on day 3, which mainly distributed in the tumor core. More necrosis areas were observed in Group D, especially on days 12 and 15.

**Figure 4:**
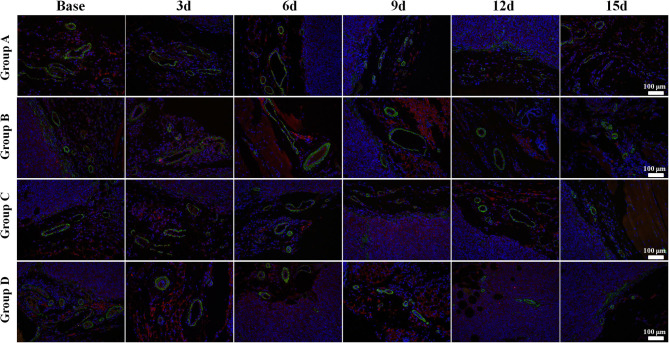
α-Smooth muscle actin (SMA) and CD31 immunofluorescent double-staining (×100) of a representative tumor section from the four groups at different time points. α-SMA was stained in green and CD31 in red. Elevated expressions of green-stained α-SMA were observed 3 days after treatment in both Groups B and D. The vessel maturity index rose to a peak on day 6 and 9, respectively, and returned to a lower level afterward in Groups B and D. No meaningful changes were observed in Groups A and C over time.

Regarding the double-staining section of α-SMA and CD31 (Figure 4), elevated expressions of green-stained α-SMA were observed 3 days after treatment in both Groups B and D. The VMI rose to a peak on day 6 and day 9, respectively, and returned to a lower level afterward in Groups B and D. No meaningful changes were observed in Groups A and C over time.

**Figure 5:**
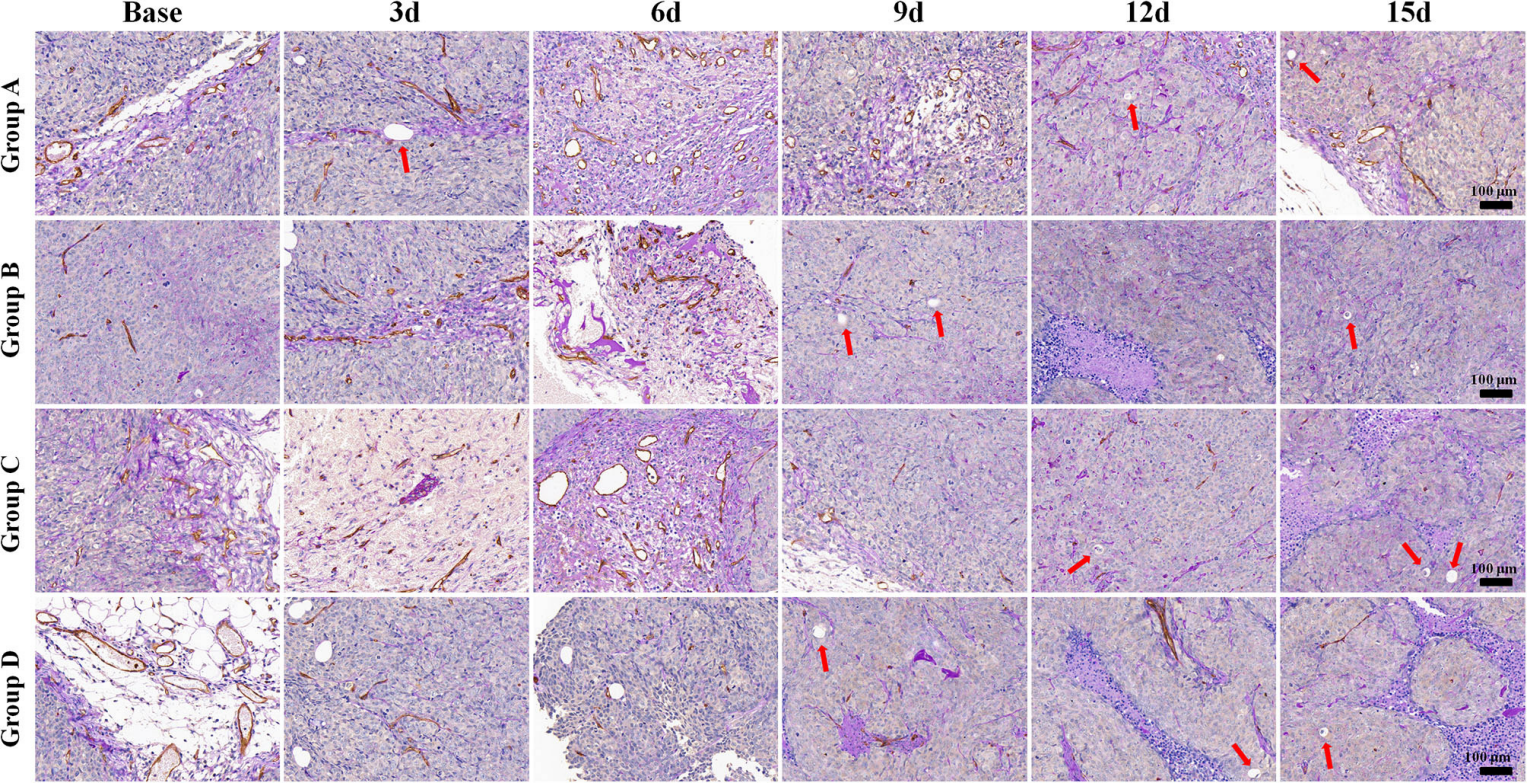
Periodic acid-Schiff (PAS) and CD31 immunohistochemical double-staining (×100) of a representative tumor section from the four groups at different time points. Both Groups B and D showed a decreased trend in microvessel density after treatment, with increased vasculogenic mimicry number in Group B. However, the numbers declined in Groups C and D 6 days after administration. Both Groups B and D showed a decreased trend in microvessel density after treatment. The number of vasculogenic mimicry in Group B increased in different speeds with time. However, downward trends were observed in Groups C and D 6 days after administration. No significant change was observed in Group A regarding microvessel density and vasculogenic mimicry.

Regarding the double-staining section of PAS and CD31 (Figure 5), both Groups B and D showed a decreased trend in MVD after treatment due to the antiangiogenic effect. The number of VM in Group B increased in different speeds over time. It obviously increased on day 9 after administration, indicating an alternative angiogenic mode was simultaneously stimulated due to the antiangiogenic effect. However, downward trends were observed in Groups C and D 6 days after administration, which meant that rather than bevacizumab, it was oxaliplatin that produced the inhibiting effect on VM. No significant change was observed in Group A regarding VM and MVD.

**Figure 6:**
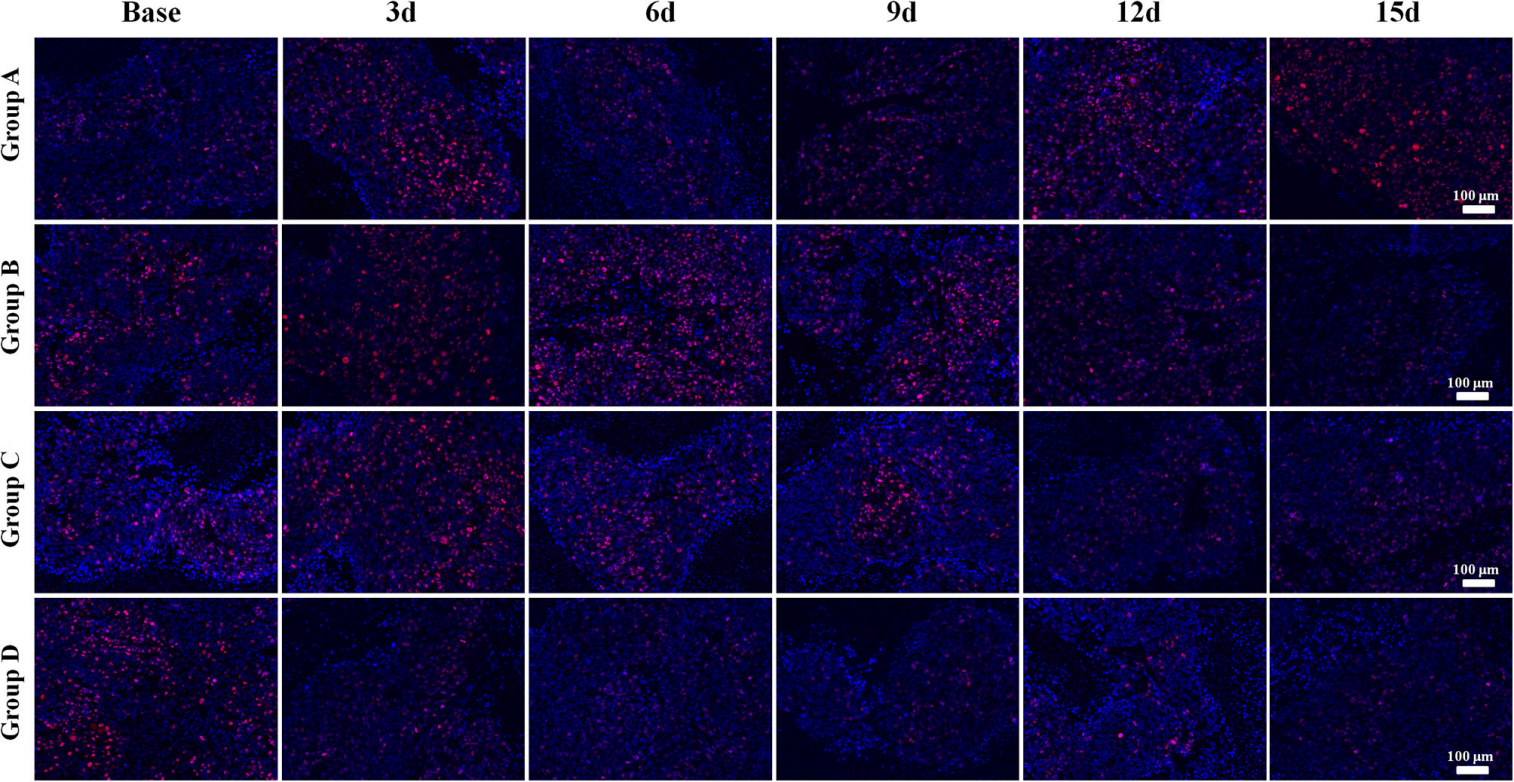
Ki-67 immunofluorescent staining (×100) of a representative tumor section from the four groups at different time points. Groups C and D showed lower Ki-67 expressions than those of the other groups, especially on days 12 and 15. Group D showed the lowest Ki-67 expression on day 15 compared to that of the other groups, indicating that combination therapy could significantly suppress the proliferation activity of tumor cells after treatment.

Group D showed the lowest Ki-67 expression on day 15 compared to the other groups, indicating that the combination therapy could have the most remarkable suppressing effect on tumor cell proliferation activity (Figure 6).

**Figure 7:**
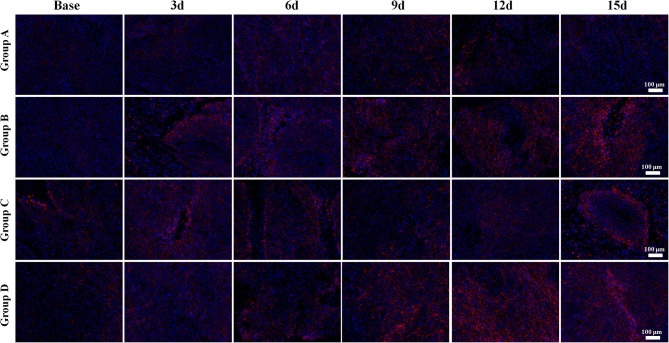
E-cadherin immunofluorescent staining (×100) of a representative tumor section from the four groups at different time points. Bright red-stained E-cadherin was the most obvious in Group D in the late time points. Groups B and C also showed an increase of E-cadherin expression after treatment. The E-cadherin expression rapidly increased in both Groups B and D, especially in Group D, indicating more stable intercellular connectivity and lower likelihood of epithelial–mesenchymal transition after treatment. A mild increase was also observed in Group C, but a slow decline was observed in Group A.

The E-cadherin expression rapidly increased in both Groups B and D, especially in Group D, indicating more stable intercellular connectivity and lower likelihood of EMT after treatment. A mild increase was also observed in Group C, but a slow decline was observed in Group A with no statistical difference (Figure 7).

### Correlation Results

The correlation coefficients between imaging and pathological indicators are listed in Table 5. In assessing tumor angiogenesis, *f*-value had the highest correlation with MVD (*r* = 0.784, *P* < 0.001), followed by D^*^ and *D*-values. *D*-value showed the strongest correlation with VM (*r* = −0.741, *P* < 0.001), followed by *f* and D^*^ value. In reflecting cell proliferation activity, *D*-value was most strongly correlated with Ki-67 (*r* = −0.781, *P* < 0.001), followed by D^*^ and *f*-value. In assessing metastatic potential of tumor cells, D^*^ value was most highly correlated with E-cadherin staining (*r* = 0.799, *P* < 0.001), followed by *f* and *D*-value. In predicting tumor growth, *D*-value had the highest negative correlation with tumor volume (*r* = −0.662, *P* < 0.001), followed by D^*^ and *f*-value.

**Table 5:** Pearson correlation coefficients between imaging and pathological parameters.

	***D***		D*		***f***	
	***r***	***P***	***r***	***P***	***r***	***P***
Tumor volumes (mm^3^)	−0.662	0.001	0.531	0.002	0.516	0.003
VMI (%)	−0.342	0.056	0.653	0.001	0.606	0.001
MVD	−0.257	0.155	0.614	0.001	0.784	0.001
VM	−0.741	0.001	0.36	0.043	0.464	0.007
Ki-67 (%)	−0.781	0.001	0.398	0.024	0.289	0.109
E-cadherin (%)	0.344	0.054	0.722	0.001	0.637	0.001

*VMI, vascular maturity index; MVD, microvessel density; VM, vasculogenic mimicry*.

## Discussion

In this study, we continuously monitored the tumor cellularity and microcirculation perfusion changes on a colon cancer model during antiangiogenesis combined with oxaliplatin treatment by IVIM-DWI. We found the combination therapy had the most significant inhibiting effect on tumor growth. The formation of vasculogenic mimicry was stimulated by single bevacizumab treatment, but was suppressed to a certain extent by oxaliplatin treatment. *D*-value significantly correlated with vasculogenic mimicry and Ki-67, while D^*^ and *f*-values showed positive correlations with microvessel density and E-cadherin. The vascular normalization and concomitant microenvironment changes can be monitored *in vivo* by the noninvasive IVIM-DWI technique.

The tumor growth was most inhibited in Group D with the tumor inhibition rate of 68.6% on day 15, followed by oxaliplatin and bevacizumab monotherapy. Besides, no obvious abnormal reaction or death occurred in the mice in the combination setting. Both pathological staining and conventional MRI suggested a large area of necrosis and decreased Ki-67 expression across the tumors at a molecular level. The higher Ki-67 expression had been reported to be correlated with a higher risk of metastasis and recurrence, poorer clinical and pathological response, and worse survival (24). It has been widely used in clinical practice as an ideal predictive and prognostic marker for cancer treatment. The lowering of Ki-67 expression by the treatment would indicate improved outcome. Moreover, the tumor invasiveness was also decreased in Group D due to an increased expression of E-cadherin, an indicator of EMT. These results are all supportive of significant improvement by bevacizumab combined with oxaliplatin therapy against single-agent therapy in colon cancer treatment. In pathology, we found that bevacizumab can improve the pericyte coverage indicated by α-SMA and promote tumor vessel maturity. Compared with the baseline, the higher VMIs during 3–12 days after bevacizumab treatment prompted a transient time window of vascular normalization. A temporary increase in blood perfusion and oxygen would relieve drug resistance resulting from tumor hypoxia. The reduced vessel permeability and interstitial pressure would further improve drug delivery and allow oxaliplatin to penetrate more deeply into the core, so that it fully inhibits tumor growth.

Hypoxia has emerged as a crucial factor in tumor pathophysiology and closely correlated with tumor invasiveness and formation of new angiogenic modes. In this study, we found one important phenomenon that VM significantly increased after bevacizumab treatment though MVD was reduced due to the antiangiogenic effect, which may account for the failure of bevacizumab monotherapy. However, the number of VM was significantly reduced in the single or combined oxaliplatin scheme, suggesting an inhibited effect on VM by a chemotherapeutic drug. This may evidence the synergistic effect of bevacizumab in the combined setting. Essentially, the VM passage is reconstructed from the crowded tumor cells due to insufficient blood supply, which are sensitive to oxaliplatin. This may imply the great value of research on therapeutic drugs that target VM formation in the future.

The tumor volume of each treatment group was much smaller than that of the control group after treatment, suggesting a considerable tumor-suppressive effect. However, evaluation on tumor volume often lags behind functional changes such as tumor perfusion and diffusion after treatment. In this study, we used IVIM-DWI to dynamically monitor the microenvironmental changes and found that both diffusion and pseudo-diffusion parameters demonstrated significant changes than tumor volumes as early as day 3 after treatment. *D*-value reflects the diffusion dynamics of water molecule, which may be limited in the crowded tumor cellularity with a high proliferative activity. Increased Ki-67 expression suggested higher cellularity and active vascular hyperplasia (25). In this study, *D*-value revealed negative and moderate correlations with tumor volume (*r* = −0.662) and Ki-67 (*r* = −0.781), indicating *D*-value can be used to predict treatment efficacy. Furthermore, both D^*^ and *f*-values shared a similar trend with VMI during day 3 to day 12 after anti-vascular treatment and had high correlations with VMI (*r* = 0.653 and 0.606) and MVD (*r* = 0.784 and 0.614), indicating that D^*^ and *f* values can be used to monitor the process of vascular normalization based on perfusion changes. Interestingly, *D*-value rather than D^*^ or *f*-value showed the highest correlation with VM. Though VM is a kind of vessel-like structure, its formation is closely correlated with tumor hypoxia and crowded cellularity and thus can be reflected by *D*-value. On the other hand, D^*^ and *f*-values had higher correlations with E-cadherin. Previous study had reported that tumor hypoxia under lower perfusion induces EMT in several types of cancer including prostate cancer, oral cancer, and breast cancer (26). An increased tumor perfusion under vascular normalization will significantly enhance E-cadherin expression and thus reduce tumor invasiveness.

There are some limitations in this study. First, hypoxia is an important characteristic of tumor microenvironment. However, we have not performed a hypoxia imaging which was introduced by Shi et al. (27). Second, we have not found a correlation between VM and E-cadherin during treatment, though they had been reported closely correlated in a hypoxia microenvironment. The role of VM and E-cadherin in an invasive microenvironment and the underlying molecular mechanisms should be further explored.

In conclusion, IVIM-DWI can well detect the microenvironment changes based on diffusion and perfusion characteristics and reflect the efficacy of cancer treatment. From the study, bevacizumab is confirmed to induce vascular normalization and have a significant inhibitory effect on MVD but could stimulate the formation of VM that has a close relationship with tumor invasiveness. The stimulation of VM can be reversed by the treatment of oxaliplatin to a certain extent. D^*^ and *f*-values are able to predict the tumor invasiveness, while D is superior in reflecting vasculogenic mimicry and Ki-67 expression.

## Data Availability Statement

The raw data supporting the conclusions of this article will be made available by the authors, without undue reservation.

## Ethics Statement

The animal study was reviewed and approved by Institutional Animal Ethics Committee of Jinan University. Written informed consent was obtained from the owners for the participation of their animals in this study.

## Author Contributions

HH, SZ, and CX contributed to conceptualization. ZL and JL contributed to the animal experiments. CP and WD contributed to MRI examinations. JL and ZL contributed to pathological analysis. JL contributed to writing the original draft preparation. CX contributed to writing, reviewing, and editing. All authors contributed to the article and approved the submitted version.

## Conflict of Interest

The authors declare that the research was conducted in the absence of any commercial or financial relationships that could be construed as a potential conflict of interest.

## Abbreviations

EMT: epithelial–mesenchymal transition
HIF-1α: hypoxia-inducible factor 1α
IVIM-DWI: intravoxel incoherent motion diffusion-weighted imaging
MRI: magnetic resonance imaging
MVD: microvessel density
VEGF: vascular endothelial growth factor
VM: vasculogenic mimicry
VMI: vessel maturity index.
